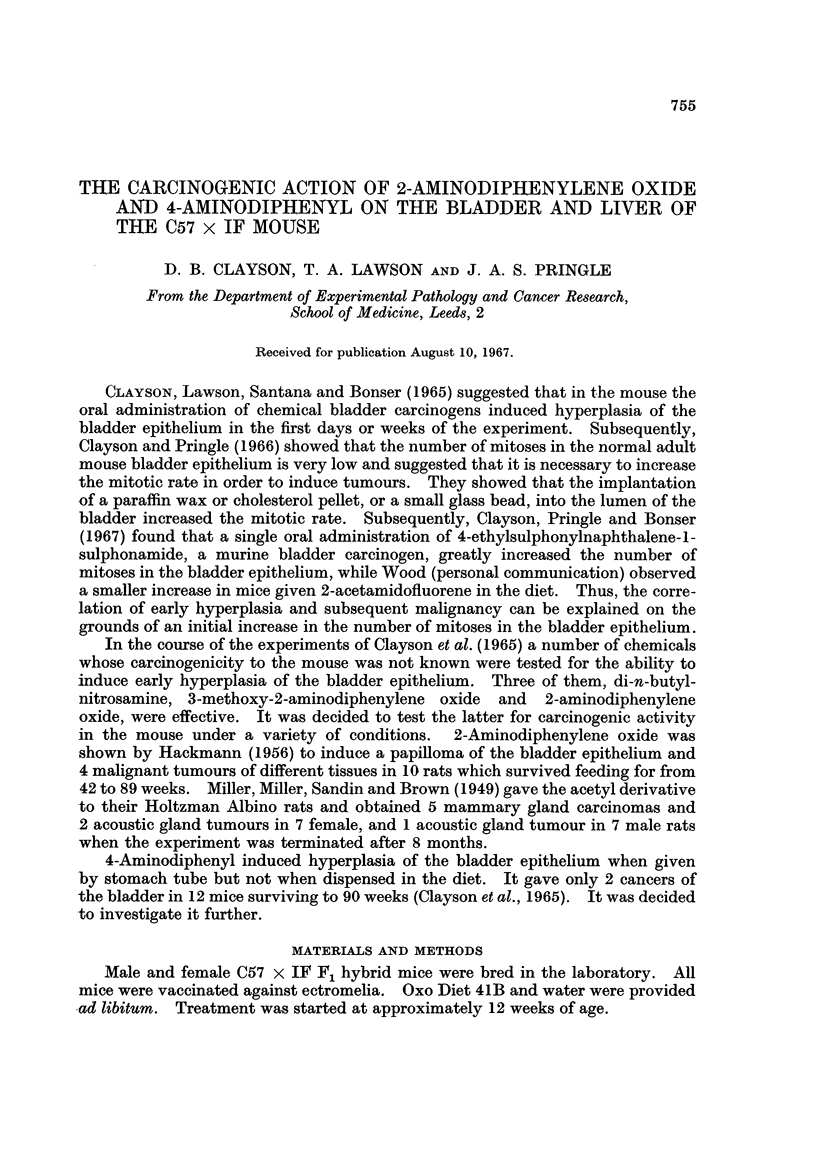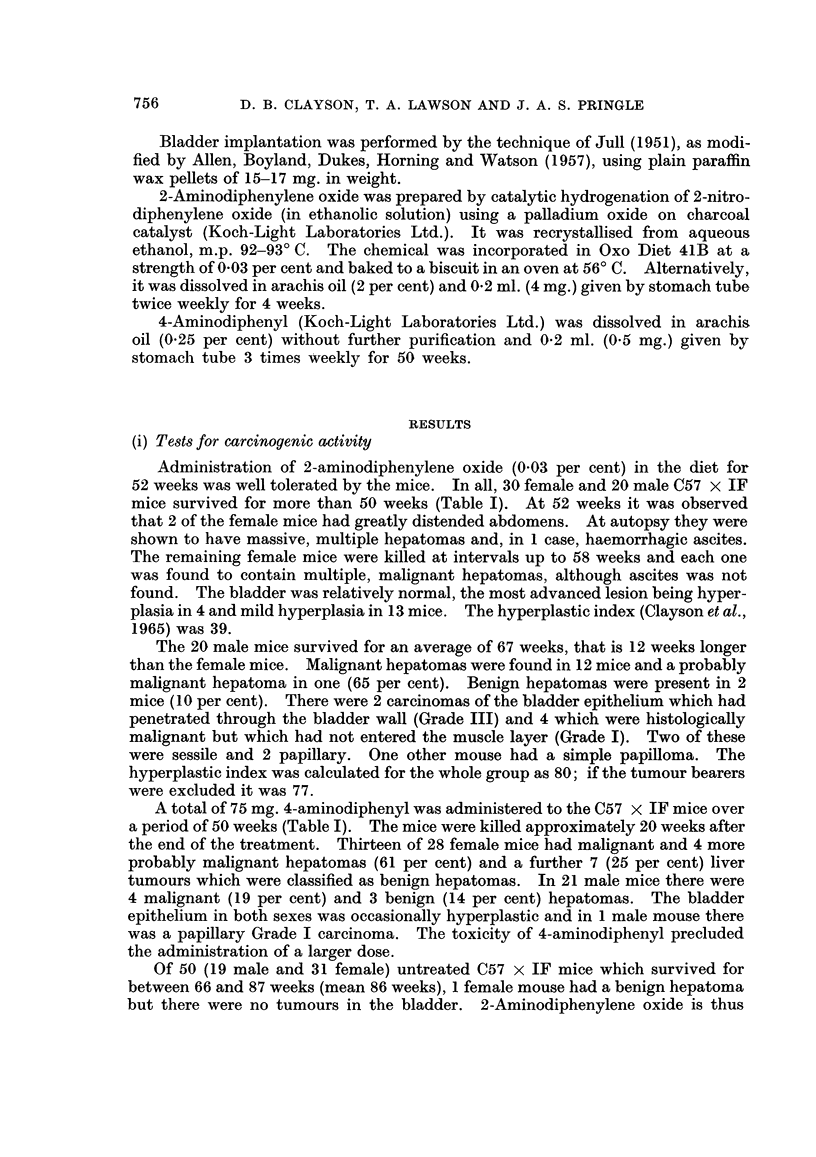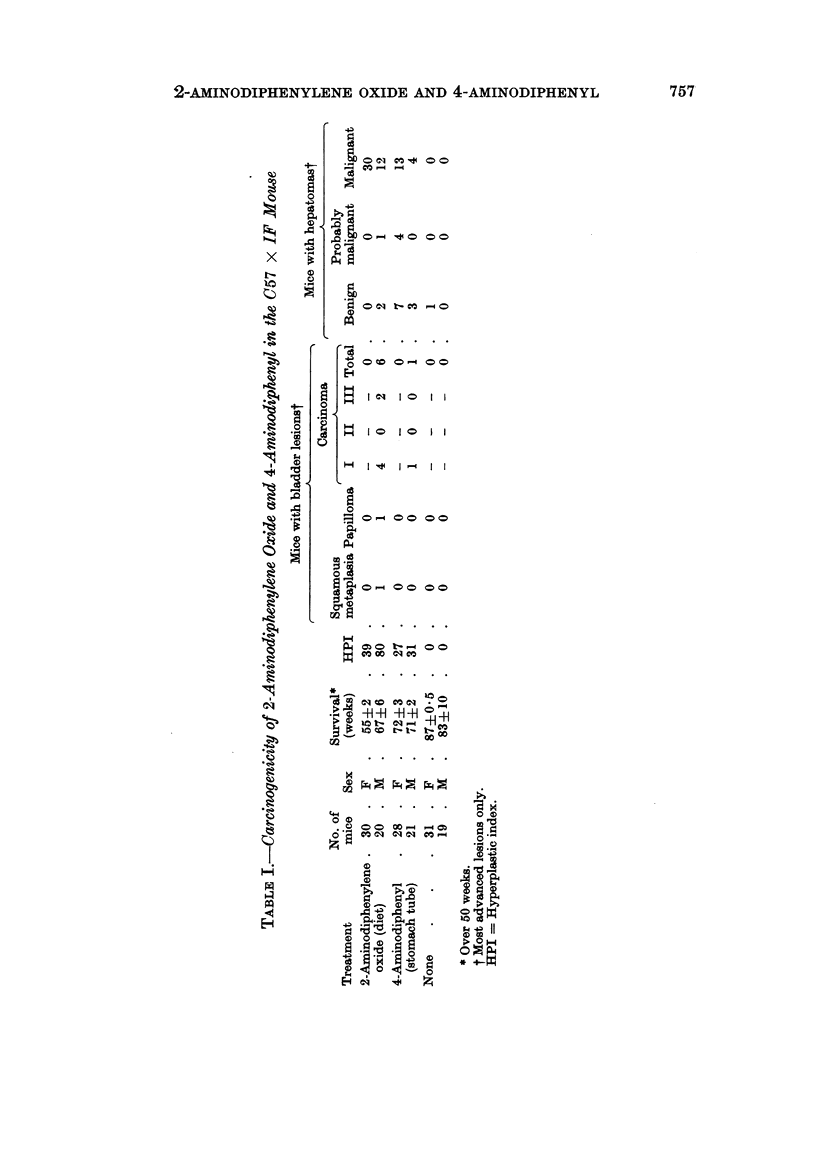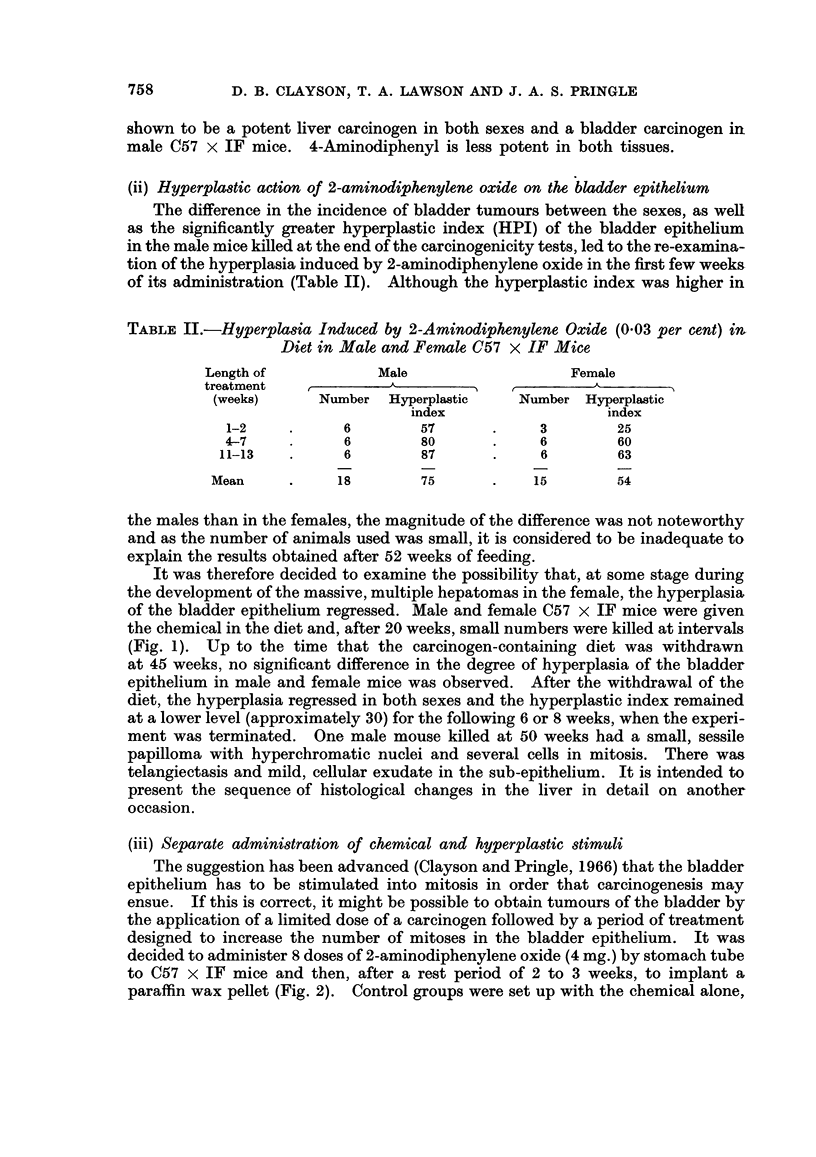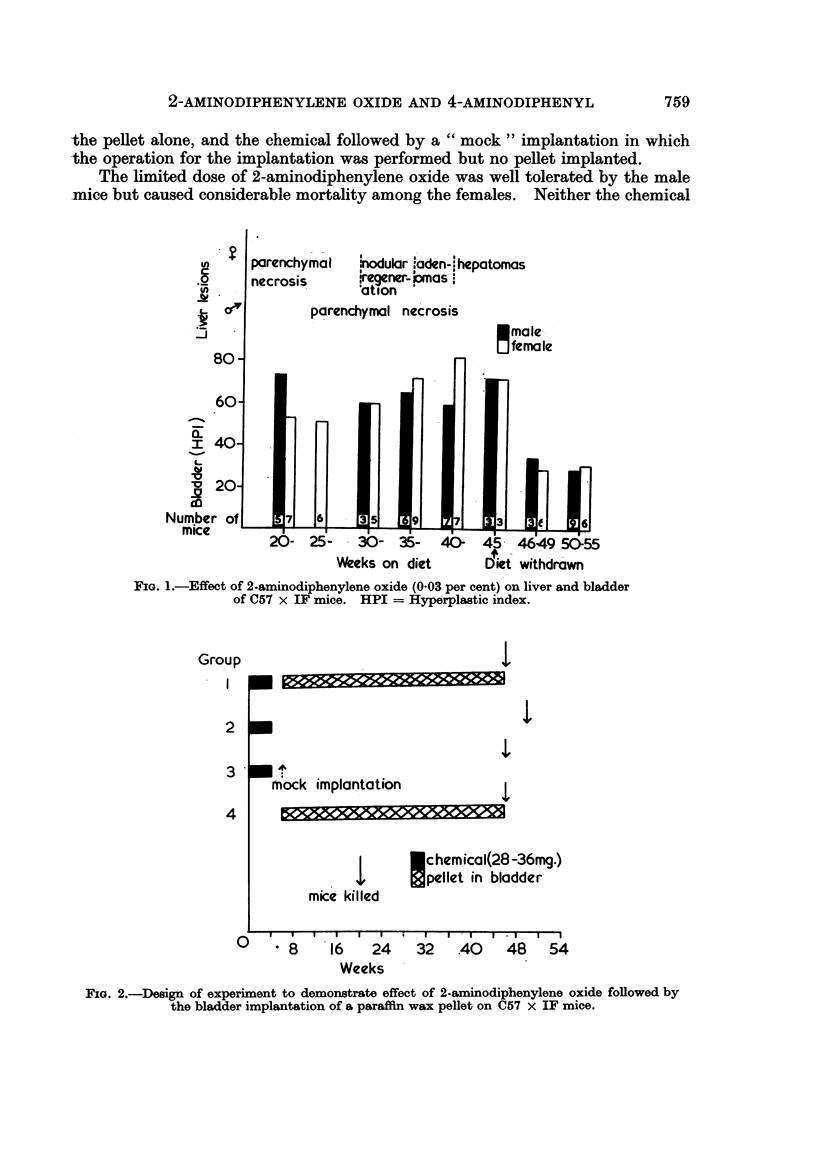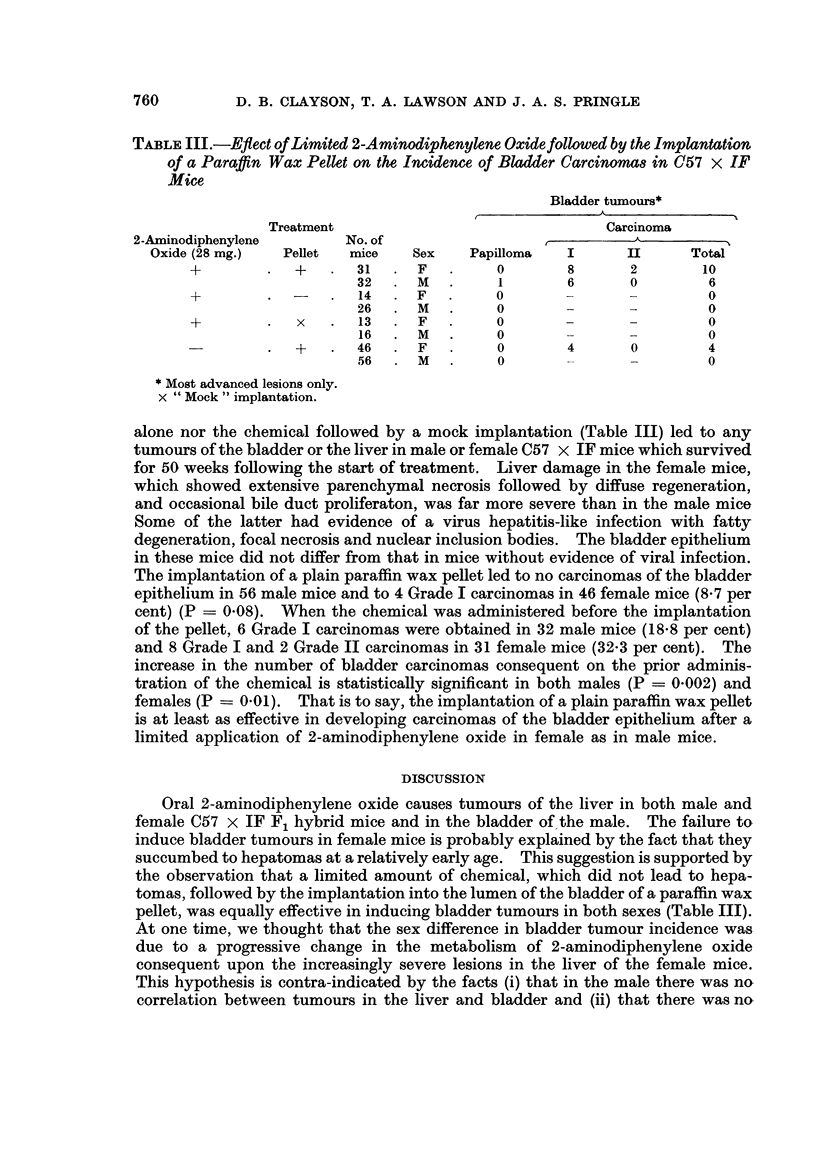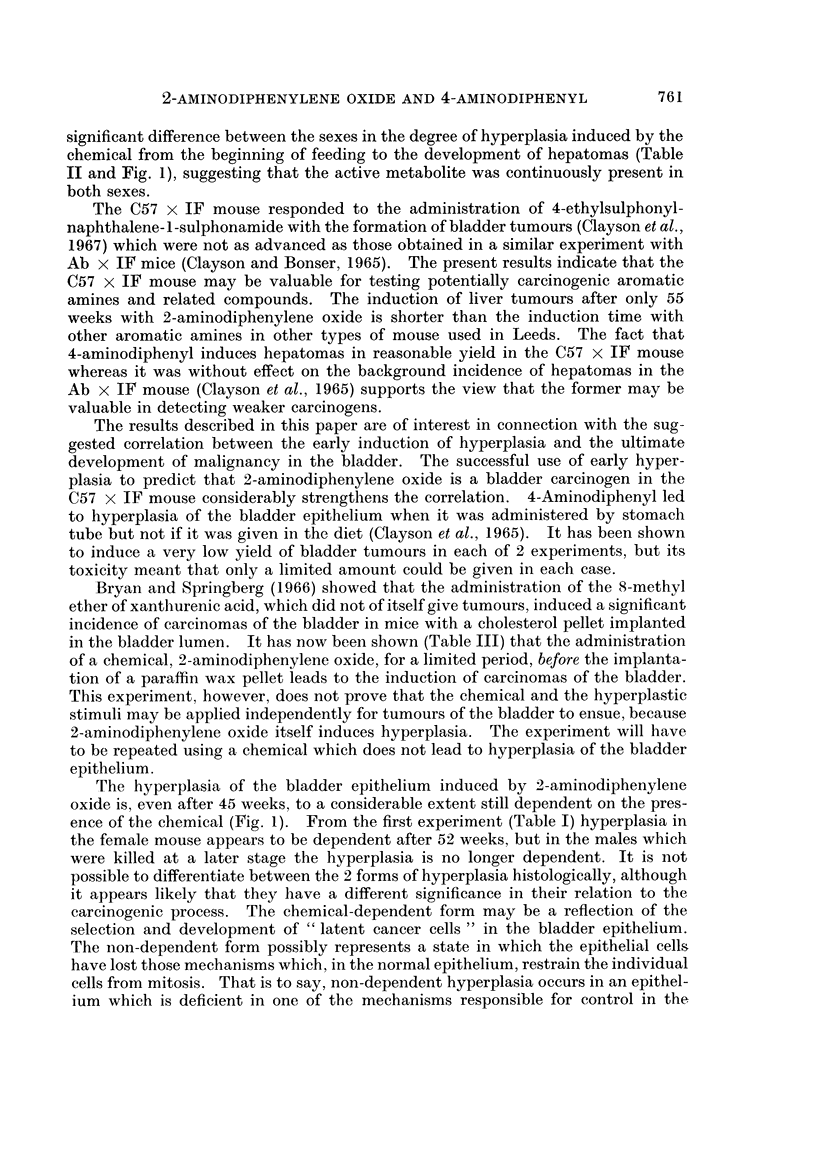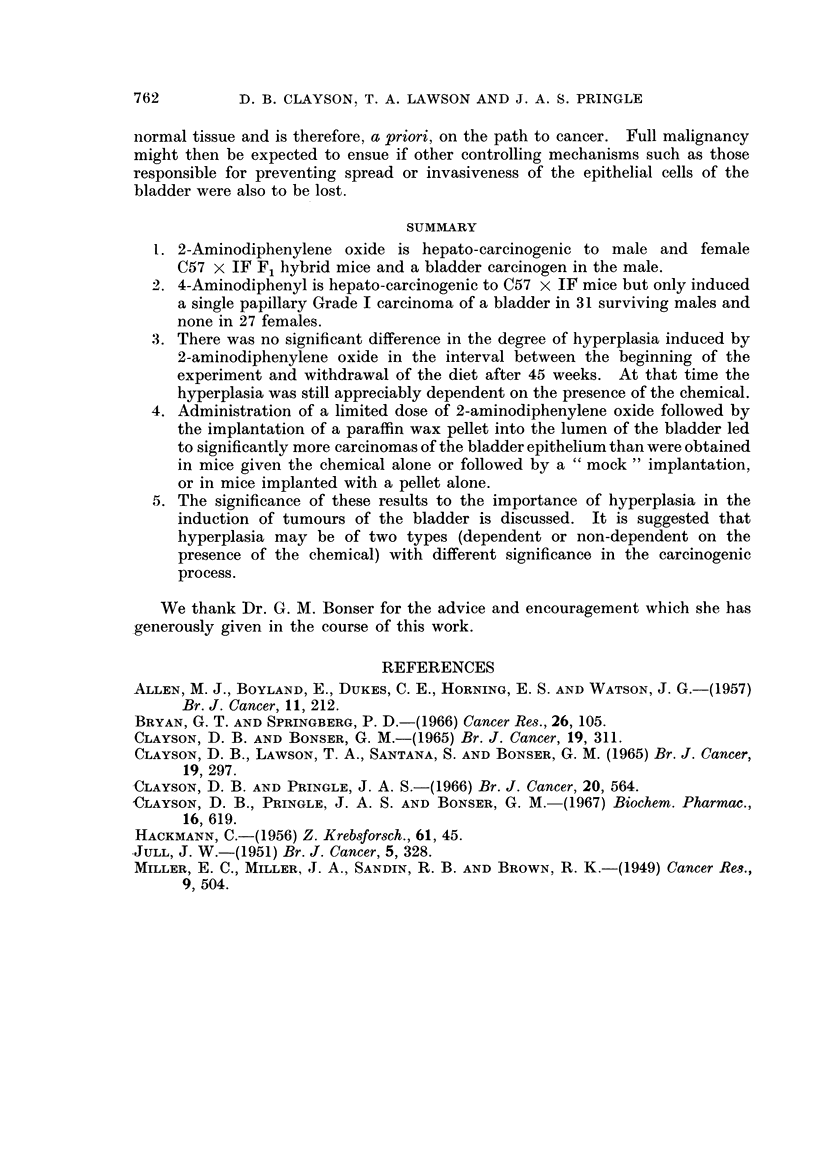# The carcinogenic action of 2-aminodiphenylene oxide and 4-aminodiphenyl on the bladder and liver of the C57 X IF mouse.

**DOI:** 10.1038/bjc.1967.88

**Published:** 1967-12

**Authors:** D. B. Clayson, T. A. Lawson, J. A. Pringle


### THE CARCINOGENIC ACTION OF 2-AMINODIPHENYLENE OXIDE AND 4-AMINODIPHENYL ON THE BLADDER AND LIVER OF THE C57 × IF MOUSE

D. B. CLAYSON, T. A. LAWSON AND J. A. S. PRINGLE

*From the Department of Experimental Pathology and Cancer Research, School of Medicine, Leeds, 2*

Received for publication August 10, 1967.

Clayson, Lawson, Santana and Bonser (1965) suggested that in the mouse the oral administration of chemical bladder carcinogens induced hyperplasia of the bladder epithelium in the first days or weeks of the experiment. Subsequently, Clayson and Pringle (1966) showed that the number of mitoses in the normal adult mouse bladder epithelium is very low and suggested that it is necessary to increase the mitotic rate in order to induce tumours. They showed that the implantation of a paraffin wax or cholesterol pellet, or a small glass bead, into the lumen of the bladder increased the mitotic rate. Subsequently, Clayson, Pringle and Bonser (1967) found that a single oral administration of 4-ethylsulphonylnaphthalene-1-sulphonamide, a murine bladder carcinogen, greatly increased the number of mitoses in the bladder epithelium, while Wood (personal communication) observed a smaller increase in mice given 2-acetamidofluorene in the diet. Thus, the correlation of early hyperplasia and subsequent malignancy can be explained on the grounds of an initial increase in the number of mitoses in the bladder epithelium.

In the course of the experiments of Clayson *et al.* (1965) a number of chemicals whose carcinogenicity to the mouse was not known were tested for the ability to induce early hyperplasia of the bladder epithelium. Three of them, di-*n*-butylnitrosamine, 3-methoxy-2-aminodiphenylene oxide and 2-aminodiphenylene oxide, were effective. It was decided to test the latter for carcinogenic activity in the mouse under a variety of conditions. 2-Aminodiphenylene oxide was shown by Hackmann (1956) to induce a papilloma of the bladder epithelium and 4 malignant tumours of different tissues in 10 rats which survived feeding for from 42 to 89 weeks. Miller, Miller, Sandin and Brown (1949) gave the acetyl derivative to their Holtzman Albino rats and obtained 5 mammary gland carcinomas and 2 acoustic gland tumours in 7 female, and 1 acoustic gland tumour in 7 male rats when the experiment was terminated after 8 months.

4-Aminodiphenyl induced hyperplasia of the bladder epithelium when given by stomach tube but not when dispensed in the diet. It gave only 2 cancers of the bladder in 12 mice surviving to 90 weeks (Clayson *et al.*, 1965). It was decided to investigate it further.

### MATERIALS AND METHODS

Male and female C57 × IF $F_1$ hybrid mice were bred in the laboratory. All mice were vaccinated against ectromelia. Oxo Diet 41B and water were provided *ad libitum*. Treatment was started at approximately 12 weeks of age.

Bladder implantation was performed by the technique of Jull (1951), as modified by Allen, Boyland, Dukes, Horning and Watson (1957), using plain paraffin wax pellets of 15–17 mg. in weight.

2-Aminodiphenylene oxide was prepared by catalytic hydrogenation of 2-nitrodiphenylene oxide (in ethanolic solution) using a palladium oxide on charcoal catalyst (Koch-Light Laboratories Ltd.). It was recrystallised from aqueous ethanol, m.p. 92–93° C. The chemical was incorporated in Oxo Diet 41B at a strength of 0·03 per cent and baked to a biscuit in an oven at 56° C. Alternatively, it was dissolved in arachis oil (2 per cent) and 0·2 ml. (4 mg.) given by stomach tube twice weekly for 4 weeks.

4-Aminodiphenyl (Koch-Light Laboratories Ltd.) was dissolved in arachis oil (0·25 per cent) without further purification and 0·2 ml. (0·5 mg.) given by stomach tube 3 times weekly for 50 weeks.

### RESULTS

### (i) *Tests for carcinogenic activity*

Administration of 2-aminodiphenylene oxide (0·03 per cent) in the diet for 52 weeks was well tolerated by the mice. In all, 30 female and 20 male C57 × IF mice survived for more than 50 weeks (Table I). At 52 weeks it was observed that 2 of the female mice had greatly distended abdomens. At autopsy they were shown to have massive, multiple hepatomas and, in 1 case, haemorrhagic ascites. The remaining female mice were killed at intervals up to 58 weeks and each one was found to contain multiple, malignant hepatomas, although ascites was not found. The bladder was relatively normal, the most advanced lesion being hyperplasia in 4 and mild hyperplasia in 13 mice. The hyperplastic index (Clayson *et al.*, 1965) was 39.

The 20 male mice survived for an average of 67 weeks, that is 12 weeks longer than the female mice. Malignant hepatomas were found in 12 mice and a probably malignant hepatoma in one (65 per cent). Benign hepatomas were present in 2 mice (10 per cent). There were 2 carcinomas of the bladder epithelium which had penetrated through the bladder wall (Grade III) and 4 which were histologically malignant but which had not entered the muscle layer (Grade I). Two of these were sessile and 2 papillary. One other mouse had a simple papilloma. The hyperplastic index was calculated for the whole group as 80; if the tumour bearers were excluded it was 77.

A total of 75 mg. 4-aminodiphenyl was administered to the C57 × IF mice over a period of 50 weeks (Table I). The mice were killed approximately 20 weeks after the end of the treatment. Thirteen of 28 female mice had malignant and 4 more probably malignant hepatomas (61 per cent) and a further 7 (25 per cent) liver tumours which were classified as benign hepatomas. In 21 male mice there were 4 malignant (19 per cent) and 3 benign (14 per cent) hepatomas. The bladder epithelium in both sexes was occasionally hyperplastic and in 1 male mouse there was a papillary Grade I carcinoma. The toxicity of 4-aminodiphenyl precluded the administration of a larger dose.

Of 50 (19 male and 31 female) untreated C57 × IF mice which survived for between 66 and 87 weeks (mean 86 weeks), 1 female mouse had a benign hepatoma but there were no tumours in the bladder. 2-Aminodiphenylene oxide is thus

TABLE I.—*Carcinogenicity of 2-Aminodiphenylene Oxide and 4-Aminodiphenyl in the C57 × IF Mouse*

| Treatment | No. of mice | Sex | Survival* (weeks) | HPI | Mice with bladder lesions† | | | | | | Mice with hepatomas† | | |
|---|---|---|---|---|---|---|---|---|---|---|---|---|---|
| | | | | | Squamous metaplasia | Papilloma | Carcinoma | | | | Benign | Probably malignant | Malignant |
| | | | | | | | I | II | III | Total | | | |
| 2-Aminodiphenylene oxide (diet) | 30 | F | 55±2 | 39 | 0 | 0 | – | – | – | 0 | 0 | 0 | 30 |
| | 20 | M | 67±6 | 80 | 1 | 1 | 4 | 0 | 2 | 6 | 2 | 1 | 12 |
| 4-Aminodiphenyl (stomach tube) | 28 | F | 72±3 | 27 | 0 | 0 | – | – | – | 0 | 7 | 4 | 13 |
| | 21 | M | 71±2 | 31 | 0 | 0 | 1 | 0 | 0 | 1 | 3 | 0 | 4 |
| None | 31 | F | 87±0·5 | 0 | 0 | 0 | – | – | – | 0 | 1 | 0 | 0 |
| | 19 | M | 83±10 | 0 | 0 | 0 | – | – | – | 0 | 0 | 0 | 0 |

† Most advanced lesions only.

HPI = Hyperplastic index.

shown to be a potent liver carcinogen in both sexes and a bladder carcinogen in male C57 × IF mice. 4-Aminodiphenyl is less potent in both tissues.

(ii) *Hyperplastic action of 2-aminodiphenylene oxide on the bladder epithelium*

The difference in the incidence of bladder tumours between the sexes, as well as the significantly greater hyperplastic index (HPI) of the bladder epithelium in the male mice killed at the end of the carcinogenicity tests, led to the re-examination of the hyperplasia induced by 2-aminodiphenylene oxide in the first few weeks of its administration (Table II). Although the hyperplastic index was higher in

TABLE II.—*Hyperplasia Induced by 2-Aminodiphenylene Oxide* (0·03 *per cent*) *in Diet in Male and Female C57 × IF Mice*

| Length of treatment (weeks) | Male | | Female | |
|---|---|---|---|---|
| | Number | Hyperplastic index | Number | Hyperplastic index |
| 1–2 | 6 | 57 | 3 | 25 |
| 4–7 | 6 | 80 | 6 | 60 |
| 11–13 | 6 | 87 | 6 | 63 |
| Mean | 18 | 75 | 15 | 54 |

the males than in the females, the magnitude of the difference was not noteworthy and as the number of animals used was small, it is considered to be inadequate to explain the results obtained after 52 weeks of feeding.

It was therefore decided to examine the possibility that, at some stage during the development of the massive, multiple hepatomas in the female, the hyperplasia of the bladder epithelium regressed. Male and female C57 × IF mice were given the chemical in the diet and, after 20 weeks, small numbers were killed at intervals (Fig. 1). Up to the time that the carcinogen-containing diet was withdrawn at 45 weeks, no significant difference in the degree of hyperplasia of the bladder epithelium in male and female mice was observed. After the withdrawal of the diet, the hyperplasia regressed in both sexes and the hyperplastic index remained at a lower level (approximately 30) for the following 6 or 8 weeks, when the experiment was terminated. One male mouse killed at 50 weeks had a small, sessile papilloma with hyperchromatic nuclei and several cells in mitosis. There was telangiectasis and mild, cellular exudate in the sub-epithelium. It is intended to present the sequence of histological changes in the liver in detail on another occasion.

(iii) *Separate administration of chemical and hyperplastic stimuli*

The suggestion has been advanced (Clayson and Pringle, 1966) that the bladder epithelium has to be stimulated into mitosis in order that carcinogenesis may ensue. If this is correct, it might be possible to obtain tumours of the bladder by the application of a limited dose of a carcinogen followed by a period of treatment designed to increase the number of mitoses in the bladder epithelium. It was decided to administer 8 doses of 2-aminodiphenylene oxide (4 mg.) by stomach tube to C57 × IF mice and then, after a rest period of 2 to 3 weeks, to implant a paraffin wax pellet (Fig. 2). Control groups were set up with the chemical alone, the pellet alone, and the chemical followed by a " mock " implantation in which the operation for the implantation was performed but no pellet implanted.

The limited dose of 2-aminodiphenylene oxide was well tolerated by the male mice but caused considerable mortality among the females. Neither the chemical

FIG. 1.—Effect of 2-aminodiphenylene oxide (0·03 per cent) on liver and bladder of C57 × IF mice. HPI = Hyperplastic index.

FIG. 2.—Design of experiment to demonstrate effect of 2-aminodiphenylene oxide followed by the bladder implantation of a paraffin wax pellet on C57 × IF mice.

TABLE III.—*Effect of Limited 2-Aminodiphenylene Oxide followed by the Implantation of a Paraffin Wax Pellet on the Incidence of Bladder Carcinomas in C57 × IF Mice*

| Treatment: 2-Aminodiphenylene Oxide (28 mg.) | Treatment: Pellet | No. of mice | Sex | Bladder tumours*: Papilloma | Carcinoma: I | Carcinoma: II | Carcinoma: Total |
|---|---|---|---|---|---|---|---|
| + | + | 31 | F | 0 | 8 | 2 | 10 |
| | | 32 | M | 1 | 6 | 0 | 6 |
| + | — | 14 | F | 0 | – | – | 0 |
| | | 26 | M | 0 | – | – | 0 |
| + | × | 13 | F | 0 | – | – | 0 |
| | | 16 | M | 0 | – | – | 0 |
| — | + | 46 | F | 0 | 4 | 0 | 4 |
| | | 56 | M | 0 | – | – | 0 |

* Most advanced lesions only.

× "Mock" implantation.

alone nor the chemical followed by a mock implantation (Table III) led to any tumours of the bladder or the liver in male or female C57 × IF mice which survived for 50 weeks following the start of treatment. Liver damage in the female mice, which showed extensive parenchymal necrosis followed by diffuse regeneration, and occasional bile duct proliferaton, was far more severe than in the male mice Some of the latter had evidence of a virus hepatitis-like infection with fatty degeneration, focal necrosis and nuclear inclusion bodies. The bladder epithelium in these mice did not differ from that in mice without evidence of viral infection. The implantation of a plain paraffin wax pellet led to no carcinomas of the bladder epithelium in 56 male mice and to 4 Grade I carcinomas in 46 female mice (8·7 per cent) (P = 0·08). When the chemical was administered before the implantation of the pellet, 6 Grade I carcinomas were obtained in 32 male mice (18·8 per cent) and 8 Grade I and 2 Grade II carcinomas in 31 female mice (32·3 per cent). The increase in the number of bladder carcinomas consequent on the prior administration of the chemical is statistically significant in both males (P = 0·002) and females (P = 0·01). That is to say, the implantation of a plain paraffin wax pellet is at least as effective in developing carcinomas of the bladder epithelium after a limited application of 2-aminodiphenylene oxide in female as in male mice.

### DISCUSSION

Oral 2-aminodiphenylene oxide causes tumours of the liver in both male and female C57 × IF $F_1$ hybrid mice and in the bladder of the male. The failure to induce bladder tumours in female mice is probably explained by the fact that they succumbed to hepatomas at a relatively early age. This suggestion is supported by the observation that a limited amount of chemical, which did not lead to hepatomas, followed by the implantation into the lumen of the bladder of a paraffin wax pellet, was equally effective in inducing bladder tumours in both sexes (Table III). At one time, we thought that the sex difference in bladder tumour incidence was due to a progressive change in the metabolism of 2-aminodiphenylene oxide consequent upon the increasingly severe lesions in the liver of the female mice. This hypothesis is contra-indicated by the facts (i) that in the male there was no correlation between tumours in the liver and bladder and (ii) that there was no significant difference between the sexes in the degree of hyperplasia induced by the chemical from the beginning of feeding to the development of hepatomas (Table II and Fig. 1), suggesting that the active metabolite was continuously present in both sexes.

The C57 × IF mouse responded to the administration of 4-ethylsulphonylnaphthalene-1-sulphonamide with the formation of bladder tumours (Clayson *et al.*, 1967) which were not as advanced as those obtained in a similar experiment with Ab × IF mice (Clayson and Bonser, 1965). The present results indicate that the C57 × IF mouse may be valuable for testing potentially carcinogenic aromatic amines and related compounds. The induction of liver tumours after only 55 weeks with 2-aminodiphenylene oxide is shorter than the induction time with other aromatic amines in other types of mouse used in Leeds. The fact that 4-aminodiphenyl induces hepatomas in reasonable yield in the C57 × IF mouse whereas it was without effect on the background incidence of hepatomas in the Ab × IF mouse (Clayson *et al.*, 1965) supports the view that the former may be valuable in detecting weaker carcinogens.

The results described in this paper are of interest in connection with the suggested correlation between the early induction of hyperplasia and the ultimate development of malignancy in the bladder. The successful use of early hyperplasia to predict that 2-aminodiphenylene oxide is a bladder carcinogen in the C57 × IF mouse considerably strengthens the correlation. 4-Aminodiphenyl led to hyperplasia of the bladder epithelium when it was administered by stomach tube but not if it was given in the diet (Clayson *et al.*, 1965). It has been shown to induce a very low yield of bladder tumours in each of 2 experiments, but its toxicity meant that only a limited amount could be given in each case.

Bryan and Springberg (1966) showed that the administration of the 8-methyl ether of xanthurenic acid, which did not of itself give tumours, induced a significant incidence of carcinomas of the bladder in mice with a cholesterol pellet implanted in the bladder lumen. It has now been shown (Table III) that the administration of a chemical, 2-aminodiphenylene oxide, for a limited period, *before* the implantation of a paraffin wax pellet leads to the induction of carcinomas of the bladder. This experiment, however, does not prove that the chemical and the hyperplastic stimuli may be applied independently for tumours of the bladder to ensue, because 2-aminodiphenylene oxide itself induces hyperplasia. The experiment will have to be repeated using a chemical which does not lead to hyperplasia of the bladder epithelium.

The hyperplasia of the bladder epithelium induced by 2-aminodiphenylene oxide is, even after 45 weeks, to a considerable extent still dependent on the presence of the chemical (Fig. 1). From the first experiment (Table I) hyperplasia in the female mouse appears to be dependent after 52 weeks, but in the males which were killed at a later stage the hyperplasia is no longer dependent. It is not possible to differentiate between the 2 forms of hyperplasia histologically, although it appears likely that they have a different significance in their relation to the carcinogenic process. The chemical-dependent form may be a reflection of the selection and development of " latent cancer cells " in the bladder epithelium. The non-dependent form possibly represents a state in which the epithelial cells have lost those mechanisms which, in the normal epithelium, restrain the individual cells from mitosis. That is to say, non-dependent hyperplasia occurs in an epithelium which is deficient in one of the mechanisms responsible for control in the normal tissue and is therefore, *a priori*, on the path to cancer. Full malignancy might then be expected to ensue if other controlling mechanisms such as those responsible for preventing spread or invasiveness of the epithelial cells of the bladder were also to be lost.

SUMMARY

1. 2-Aminodiphenylene oxide is hepato-carcinogenic to male and female C57 $\times$ IF $F_1$ hybrid mice and a bladder carcinogen in the male.
2. 4-Aminodiphenyl is hepato-carcinogenic to C57 $\times$ IF mice but only induced a single papillary Grade I carcinoma of a bladder in 31 surviving males and none in 27 females.
3. There was no significant difference in the degree of hyperplasia induced by 2-aminodiphenylene oxide in the interval between the beginning of the experiment and withdrawal of the diet after 45 weeks. At that time the hyperplasia was still appreciably dependent on the presence of the chemical.
4. Administration of a limited dose of 2-aminodiphenylene oxide followed by the implantation of a paraffin wax pellet into the lumen of the bladder led to significantly more carcinomas of the bladder epithelium than were obtained in mice given the chemical alone or followed by a " mock " implantation, or in mice implanted with a pellet alone.
5. The significance of these results to the importance of hyperplasia in the induction of tumours of the bladder is discussed. It is suggested that hyperplasia may be of two types (dependent or non-dependent on the presence of the chemical) with different significance in the carcinogenic process.

We thank Dr. G. M. Bonser for the advice and encouragement which she has generously given in the course of this work.

### REFERENCES

ALLEN, M. J., BOYLAND, E., DUKES, C. E., HORNING, E. S. AND WATSON, J. G.—(1957) *Br. J. Cancer*, **11**, 212.

BRYAN, G. T. AND SPRINGBERG, P. D.—(1966) *Cancer Res.*, **26**, 105.

CLAYSON, D. B. AND BONSER, G. M.—(1965) *Br. J. Cancer*, **19**, 311.

CLAYSON, D. B., LAWSON, T. A., SANTANA, S. AND BONSER, G. M. (1965) *Br. J. Cancer*, **19**, 297.

CLAYSON, D. B. AND PRINGLE, J. A. S.—(1966) *Br. J. Cancer*, **20**, 564.

CLAYSON, D. B., PRINGLE, J. A. S. AND BONSER, G. M.—(1967) *Biochem. Pharmac.*, **16**, 619.

HACKMANN, C.—(1956) *Z. Krebsforsch.*, **61**, 45.

JULL, J. W.—(1951) *Br. J. Cancer*, **5**, 328.

MILLER, E. C., MILLER, J. A., SANDIN, R. B. AND BROWN, R. K.—(1949) *Cancer Res.*, **9**, 504.